# Deep Learning for Approaching Hepatocellular Carcinoma Ultrasound Screening Dilemma: Identification of α-Fetoprotein-Negative Hepatocellular Carcinoma From Focal Liver Lesion Found in High-Risk Patients

**DOI:** 10.3389/fonc.2022.862297

**Published:** 2022-05-26

**Authors:** Wei-bin Zhang, Si-ze Hou, Yan-ling Chen, Feng Mao, Yi Dong, Jian-gang Chen, Wen-ping Wang

**Affiliations:** ^1^ Department of Ultrasound, Zhongshan Hospital of Fudan University, Shanghai, China; ^2^ Department of Ultrasound, Zhongshan hospital of Fudan University (Xiamen Branch), Xiamen, China; ^3^ Department of Mathematical Sciences, School of Physical Sciences, University of Liverpool, Liverpool, United Kingdom; ^4^ Shanghai Key Laboratory of Multidimensional Information Processing, School of Communication & Electronic Engineering, East China Normal University, Shanghai, China

**Keywords:** deep learning, ultrasound, AFP negative, hepatocellular carcinoma, focal liver lesion, focal nodular hyperplasia, HBV infection

## Abstract

**Background:**

First-line surveillance on hepatitis B virus (HBV)-infected populations with B-mode ultrasound is relatively limited to identifying hepatocellular carcinoma (HCC) without elevated α-fetoprotein (AFP). To improve the present HCC surveillance strategy, the state of the art of artificial intelligence (AI), a deep learning (DL) approach, is proposed to assist in the diagnosis of a focal liver lesion (FLL) in HBV-infected liver background.

**Methods:**

Our proposed deep learning model was based on B-mode ultrasound images of surgery that proved 209 HCC and 198 focal nodular hyperplasia (FNH) cases with 413 lesions. The model cohort and test cohort were set at a ratio of 3:1, in which the test cohort was composed of AFP-negative HBV-infected cases. Four additional deep learning models (MobileNet, Resnet50, DenseNet121, and InceptionV3) were also constructed as comparative baselines. To evaluate the models in terms of diagnostic power, sensitivity, specificity, accuracy, confusion matrix, F1-score, and area under the receiver operating characteristic curve (AUC) were calculated in the test cohort.

**Results:**

The AUC of our model, Xception, achieved 93.68% in the test cohort, superior to other baselines (89.06%, 85.67%, 83.94%, and 78.13% respectively for MobileNet, Resnet50, DenseNet121, and InceptionV3). In terms of diagnostic power, our model showed sensitivity, specificity, accuracy, and F1-score of 96.08%, 76.92%, 86.41%, and 87.50%, respectively, and PPV, NPV, FPR, and FNR calculated from the confusion matrix were respectively 80.33%, 95.24%, 23.08%, and 3.92% in identifying AFP-negative HCC from HBV-infected FLL cases. Satisfactory robustness of our proposed model was shown based on 5-fold cross-validation performed among the models above.

**Conclusions:**

Our DL approach has great potential to assist B-mode ultrasound in identifying AFP-negative HCC from FLL found in surveillance of HBV-infected patients.

## Introduction

Liver cancer ranks as the third leading cause of cancer-related death worldwide. Hepatocellular carcinoma (HCC) is the most common primary malignancy, which accounts for about 85%–90% of all primary hepatocellular carcinoma (1). Hepatitis B virus (HBV) and hepatitis C virus (HCV) continue to be attributed as major causes of the global burden of HCC; notably, HBV-related HCC accounts for about 77% of HCC patients in China (2). For those with high risks of HCC, regular screening for early stages of HCC usually achieves a relatively good prognosis. On surveillance of HBV-infected patients, ultrasound (US) screening of the liver with/without serum α-fetoprotein (AFP) has been recommended as the initial examination in major guidelines (APASL 2017, EASL 2018, AASLD 2018, JSH 2021, China 2019) (3–8). An elevated serum AFP with the finding of liver neoplasm can easily lead to a diagnosis of HCC in patients at risk. However, elevated serum AFP was detected in only one-third of patients at any stage of HCC, and AFP-negative HCC still covers a large proportion of the whole HCC patients (9). Given the fact that most cases of benign focal liver lesions (FLLs) do not present alleviated serum AFP levels, identification of biomarker negative HCC is crucial for early clinical intervention. Therefore, cost-effective and reliable methods are required for patients at risk of AFP-negative HCC.

Conventional B-mode US has been shown to be a rapid, non-invasive, cost-effective, and widely available tool for liver neoplasm screening, while B-mode US is less accurate and sensitive at differentiating HCC from benign FLLs without AFP measurement or alleviated AFP. According to a recent meta-analysis, US alone has a low sensitivity of 63% and 45% to detect early-stage HCC in patients at risk with and without AFP detected (10). In comparison, an annual contrast-enhanced MRI/CT demonstrated superior performance to biannual US in the surveillance of early-stage HCC, and its combination with AFP was not statistically different for MRI (11, 12). There is still much space for improvement in US-based HCC surveillance. Among the benign FLLs, a hemangioma can be easily identified by US and MRI even without contrast agents (13, 14), most of which will be categorized into US-1, according to the US Liver Imaging Reporting and Data System (US LI-RADS) (15). Focal nodular hyperplasia (FNH), the second most common FLL, only behind hemangioma, shares similar presentations with AFP-negative HCC in non-contrast-enhanced imaging (US/CT/MRI) and clinical background, easily misdiagnosed especially in those at risk of HCC (16). According to the US LI-RADS, an FLL with a size over 10 mm in patients at risk for developing HCC will be categorized as US-3, which is positive in the screening process where contrast-enhanced imaging is recommended (15, 17, 18). Apparently, advanced examination methods are not suitable for individual surveillance due to high cost, risk of complications, and often empiricism on patients with negative biomarkers. There is still a need for easy-to-use screening methods with more objectivity and sensitivity to improve current US-based HCC surveillance.

The development of artificial intelligence (AI) provides an opportunity to improve the accuracy of current clinical surveillance and diagnosis strategy. It has the potential to identify liver carcinoma from benign liver lesions using US alone, which shed light on the screening of AFP-negative HCC from FLL found in high-risk populations (19, 20). As the state-of-the-art machine learning (ML) approach in the field of AI, deep learning (DL) is getting more attention in the field of medicine. However, the better accuracy of DL methods demands a relatively large sample-based model. Given enormous data generated by the first-line surveillance of HBV-infected population with US, we developed a DL model based on B-mode US images of 209 HCC and 198 FNH cases to investigate its potential in identifying AFP-negative HCC from FLL found in HBV-infected patients during surveillance.

## Materials and Methods

### Overall Design

**Figure 1:**
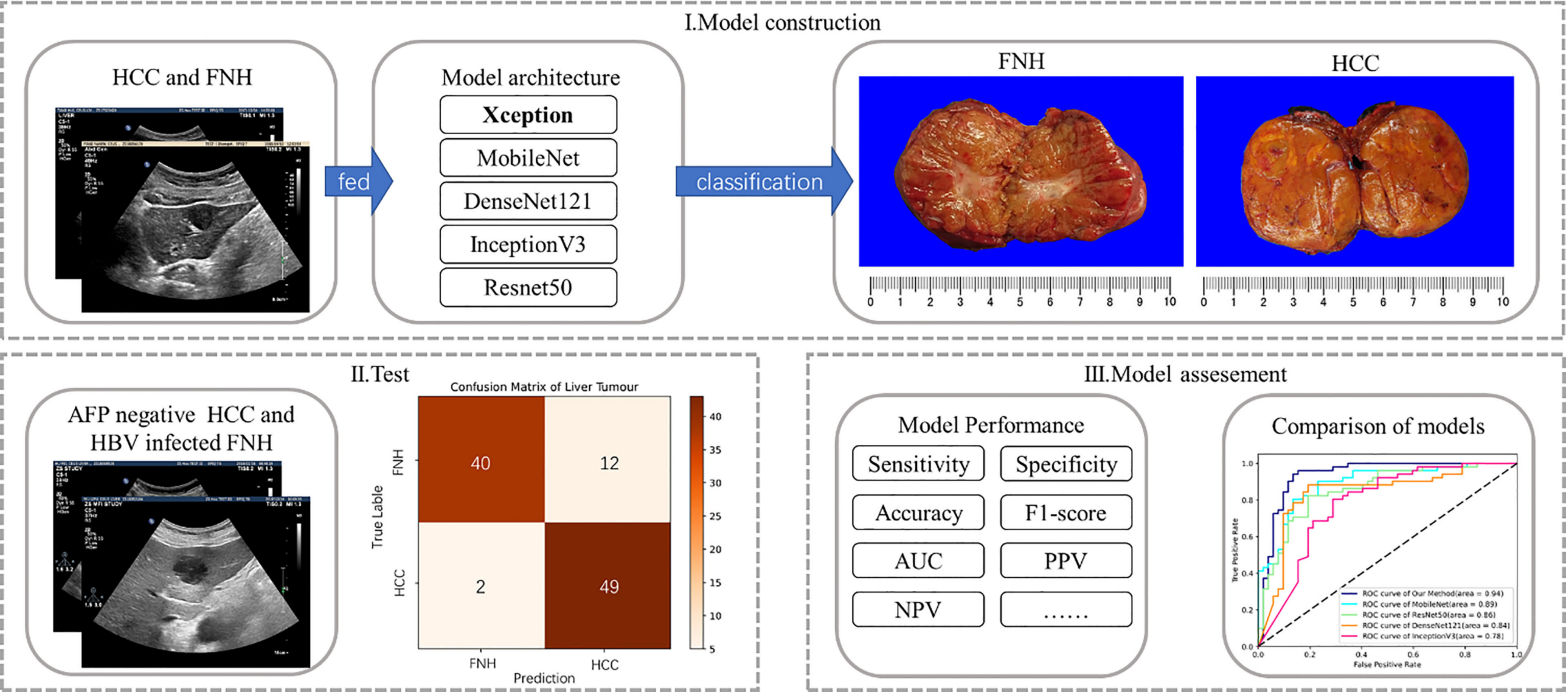
Flowchart of deep learning model construction and analysis: (i) obtained grayscale images of the model cohort were fed into five deep learning models for training and model construction; (ii) selected lesions of the test cohort with similar clinical backgrounds were tested; and (iii) the five deep learning models were assessed in terms of diagnostic performance.

To investigate the potential of the DL method based on B-mode US for differential diagnosis of AFP-negative HCC from benign FLL in HBV-infected patients, we recruited patients who presented with FLL on B-mode US on screening, all histologically confirmed by surgery. As most hemangioma presents typically on B-mode US, we selected FNH, the second most common benign FLL, as the control group, which is more difficult to differentiate from HCC solely on B-mode US. The model cohort consecutively enrolled patients with all stages of HCC or FNH regardless of AFP level, in order to obtain the most information from B-mode US images. FLLs were allocated consecutively to the test cohort according to negative serum AFP and with a history of HBV infection, with the model cohort and test cohort at a ratio of 3:1. The diagnostic performance of our proposed method was compared with that of other often used DL methods (**Figure 1**).

### Patients and Lesions

This study was approved by the Ethics Committee of the Zhongshan Hospital of Fudan University.

**Figure 2:**
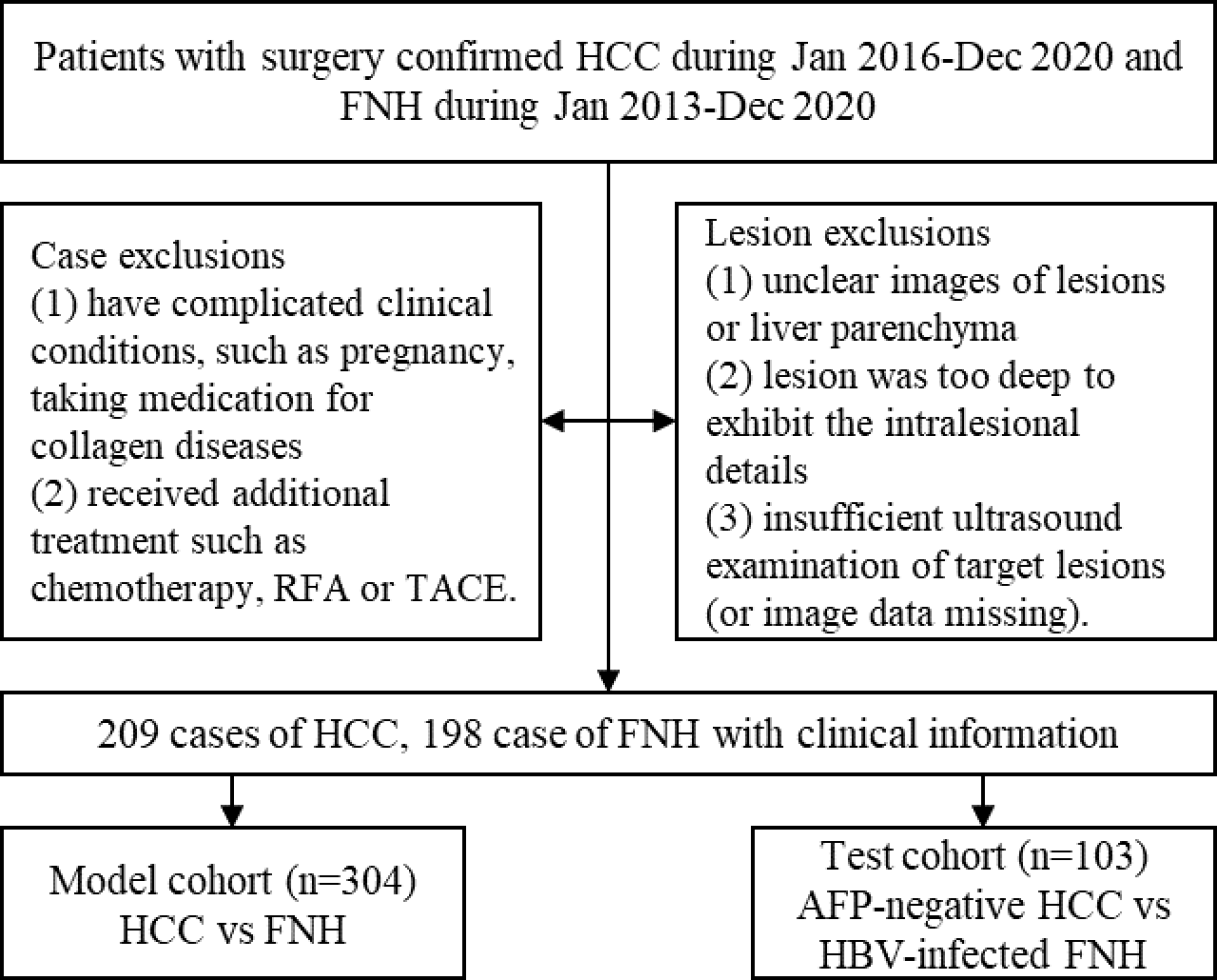
The flowchart of patient selection process. HCC, hepatocellular carcinoma; FNH, focal nodular hyperplasia.

Patients were included according to the following criteria: 1) patients with HCC and FNH were all pathologically confirmed after surgical resection; 2) all enrolled patients underwent US examination before surgery; and 3) patients with multiple lesions had pathologically confirmed ones enrolled. The exclusion criteria were as follows: 1) patients have complicated clinical conditions such as pregnancy and taking medication for collagen diseases; 2) patients received additional treatment before examination such as chemotherapy, radiofrequency ablation (RFA), or transcatheter arterial chemoembolization (TACE). Finally, 407 patients were enrolled. Four cases with multiple lesions had confirmed lesions assessed (**Figure 2**).

Clinical information within 2 weeks before surgery of the enrolled patient was collected, including age, gender, AFP, and 5 serum biomarkers of HBV (21). The threshold value for a negative AFP level was set below 20 ng/ml, and past infection of HBV was identified according to the European Association for the Study of the Liver (EASL) 2017 guideline (21).

### Image Acquisition

US B-mode images of liver lesions were obtained on iU22, EPIQ7 (Philips, Andover, MA, USA), LOGIQ E9 (GE, London, UK), Aplio 500 (Canon, Tokyo, Japan), and MyLab Twice (Esaote, Milan, Italy). An optimal slice of each lesion was selected for further analysis from the restored image sequences. The criteria of US images selection were as follows: 1) images showing lesions with liver parenchyma background and 2) with the size >1 and <10 cm. The exclusions of images were as follows: 1) unclear images of lesions or liver parenchyma; 2) lesion was too deep to exhibit intralesional details; and 3) insufficient US examination of target lesions (or image data missing). A total of 413 lesions were included (**Figure 2**).

### Setting Up the Cohorts of Model and Test

The patients were allocated to the model cohort and test cohort at a ratio of 3:1, with the ratio of HCC and FNH group at about 1:1. The model cohort consecutively enrolled patients with all stages of HCC or FNH regardless of AFP level, which was allocated to a training set and an internal validation set randomly at a ratio of 4:1 for model establishment. FLLs with negative serum AFP and a history of HBV infection were allocated consecutively to the test cohort for external validation. We set such groups as the test cohort, to see if the DL method is able to differentiate HCC from FNH with similar clinical backgrounds. The model cohort was used for training and model establishment. The test cohort was not integrated into the DL models during training.

### Model Architecture

Our proposed model is Xception, which is based on the convolutional neural network architecture.

**Figure 3:**
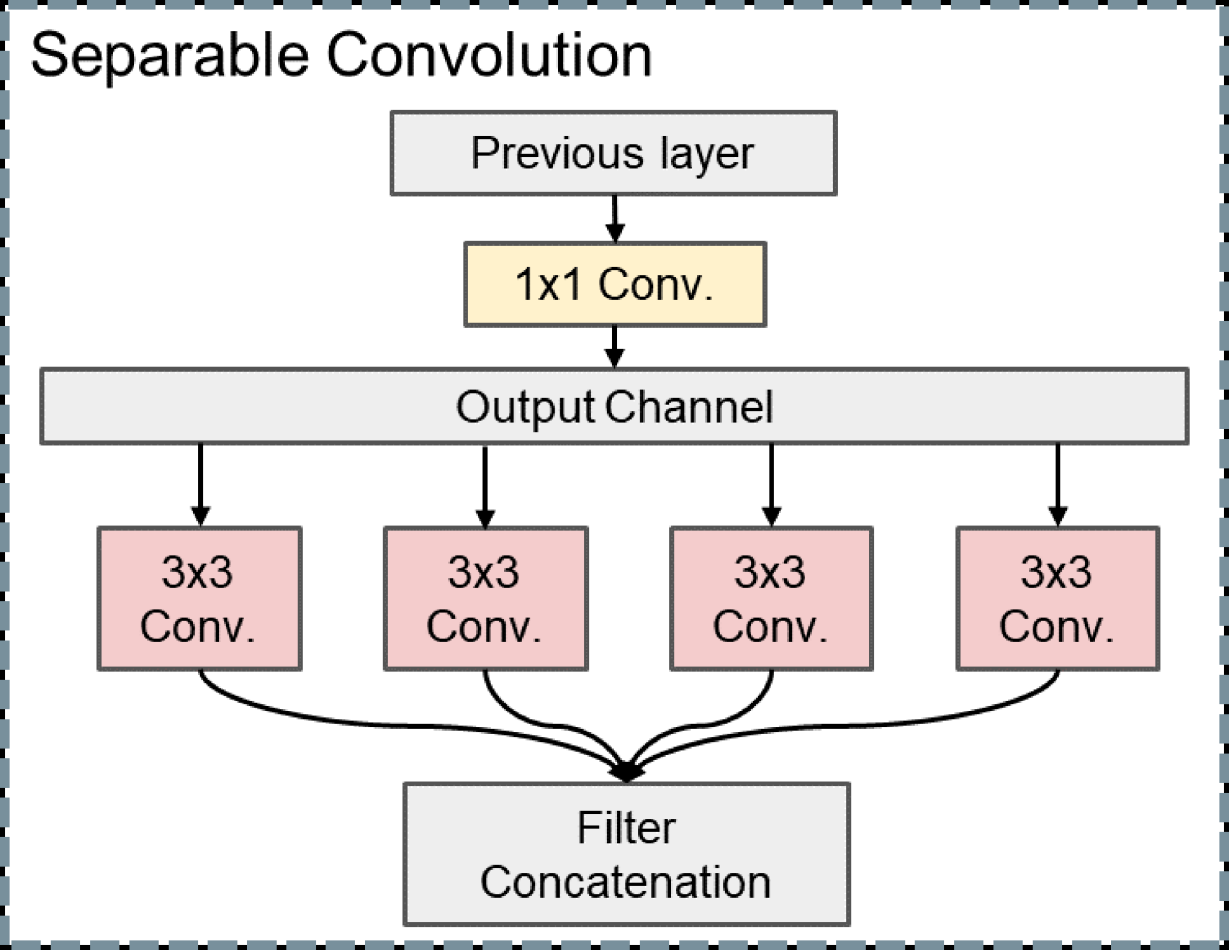
Structure of separable convolution.

When the convolutional neural network extracts the feature of our liver US images, the cross-channel cross-correlation operation and the single-channel spatial cross-correlation operation are completely separable, and the joint mapping could be detrimental. Different from other DL models, we decompose the convolution operation into separable convolution, which is a series of independent 1 × 1 cross-channel convolution and spatial convolutions operations of each channel. This separable convolution can save many parameters in the model (**Figure 3**).

**Figure 4:**
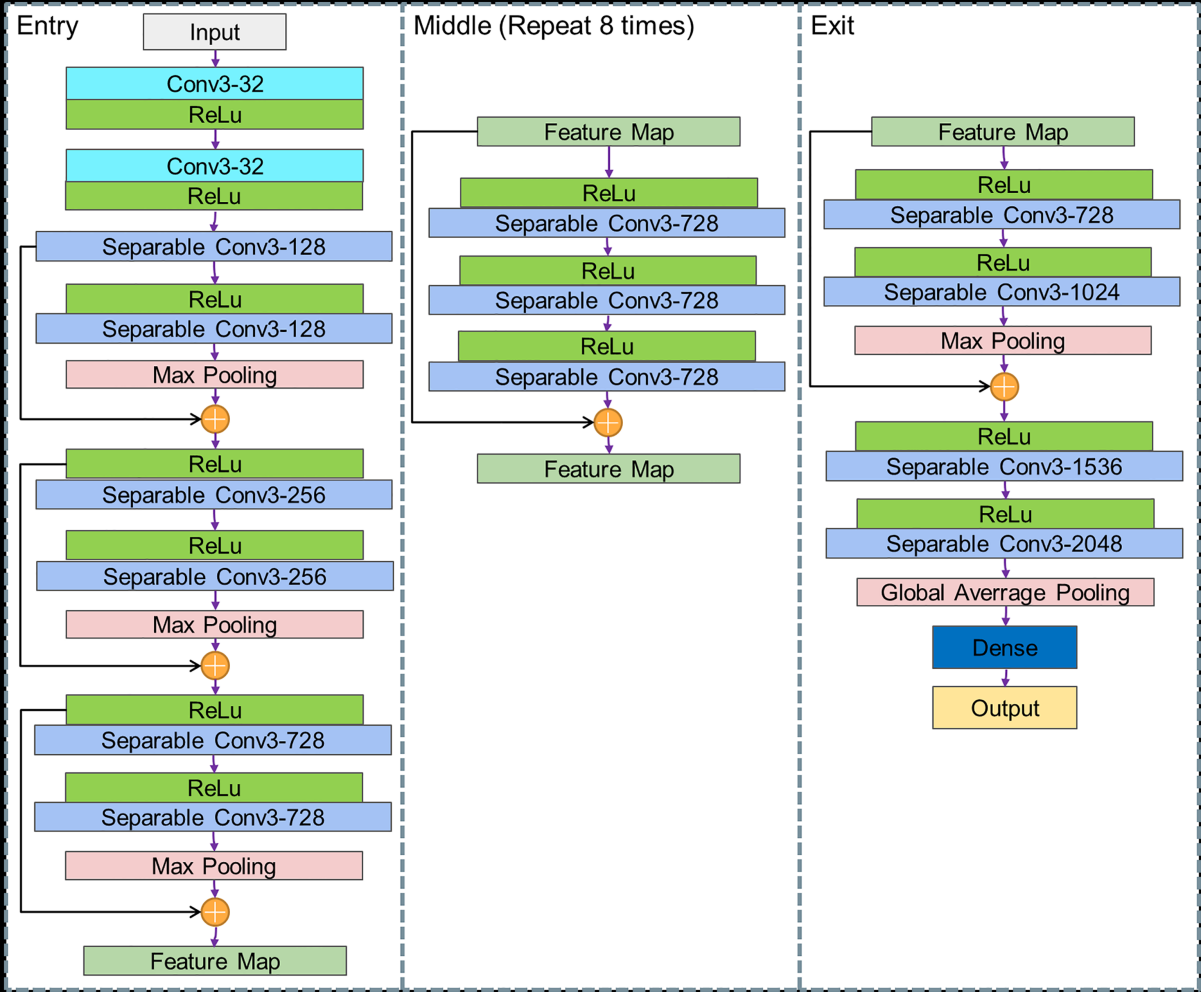
Structure of Xception.

In order to find more abstract lesions features, our Xception model uses 36 convolutional layers to form the entire DL model. Except for the first and last modules, all these modules are formed by linear residual connections based on ResNet to deepen our model. The convolutional layer is replaced with separable convolution. As shown in **Figure 4**, the entire network is divided into three parts: entry, middle, and exit.

### Model Assessment

To evaluate the classification models in terms of diagnostic power, sensitivity, specificity, accuracy, F1-score, positive predictive value (PPV), negative predictive value (NPV), false-positive rate (FPR), and false-negative rate (FNR) were calculated. Receiver operating characteristic (ROC) curves were depicted to reflect the diagnostic power in an intuitive way and to compute the area under the ROC curve (AUC). We compared the performance of our proposed DL model with the mature lightweight convolutional neural network MobileNet, the most widely used image classification model Resnet50, a well-known complex DL model with fewer parameters DenseNet121, and a SOTA multi-scale Convolutional Neural Network InceptionV3, in terms of diagnostic power.

The diagnostic performance gained from the test cohort was capped at 100 epochs of training. For comparable robustness of DL models noted above, models in 5-fold cross-validation were capped at 50 epochs. The given model cohort dataset is split into 5 number folds, where each fold is used as a validation set at some point and other folds are used as the training set. This process is repeated until each fold of the 5 folds has been used as the validation set.

## Results

### Clinical Information

A total of 407 cases were enrolled in our study, comprising 209 HCC and 198 FNH cases. All lesions included were surgically proved. The clinical information of the patients in the model cohort and test cohort are shown in **Table 1**. As such complicated cases were assembled in the test cohort (lesion without alleviated serum AFP in HBV-infected cases), a significant difference was found between the model and test cohorts with regard to HBV infection and AFP (*p* < 0.05). Age and lesion size were found relatively different in both cohorts, and we assume that this was a result of AFP-negative HCC in a small lesion, and FNH is usually found at a young age. No significant difference was found between the two cohorts with regard to factors that largely influence the diagnosis process, such as gender, lesion echogenicity, fatty liver, and liver cirrhosis (*p* ≥ 0.05).

**Table 1:** Baseline information in the model and test cohorts.

Parameters	Model	Test	*p*	HCC	FNH
Case	n = 305	n = 102	-	n = 209	n = 198
Age	44.51 ± 16.34	48.12 ± 13.68	0.000	56.24 ± 10.96	34.31 ± 11.75
Gender			0.722		
Male	214 (70.2)	73 (71.6)	–	179 (85.6)	111 (56.1)
Female	91 (29.8)	29 (28.4)	–	30 (14.4)	87 (43.9)
HBV infection	132 (43.3)	102 (100.0)	0.000	188 (90.0)	50 (25.3)
AFP ≥ 20 (ng/ml)	105 (34.4)	0	0.000	110 (52.6)	0
Lesion	n = 310	n = 103	–	n = 209	n = 204
Lesion size	45.35 ± 27.71	35.54 ± 19.94	0.004	39.95 ± 30.38	45.93 ± 21.02
≥3 cm	211 (68.1)	56 (54.4)	–	122 (58.4)	145 (71.1)
<3 cm	99 (31.9)	47 (45.6)	–	87 (41.6)	59 (28.9)
Lesion echogenicity			0.421		
Hypo-	197 (63.5)	70 (68.0)	–	135 (64.6)	132 (64.7)
Iso-	60 (19.4)	21 (20.4)	–	30 (14.4)	51 (25.0)
Hyper-	53 (17.1)	12 (11.7)	–	44 (21.1)	21 (10.3)
Liver background					
Fatty liver	49 (15.8)	17 (16.5)	0.867	24 (11.5)	42 (20.6)
Liver fibrosis	59 (19.0)	36 (35.0)	0.001	80 (38.3)	15 (7.4)
Liver cirrhosis	80 (25.8)	25 (24.3)	0.757	105 (50.2)	0

Data are presented as mean ± SD or n (%); p-value is set to <0.05 to suggest statistical difference between the model and test cohorts.

HCC, hepatocellular carcinoma; FNH, focal nodular hyperplasia; HBV, hepatitis B virus; AFP, α-fetoprotein.

### Diagnostic Performance of Deep Learning Methods

**Figure 5:**
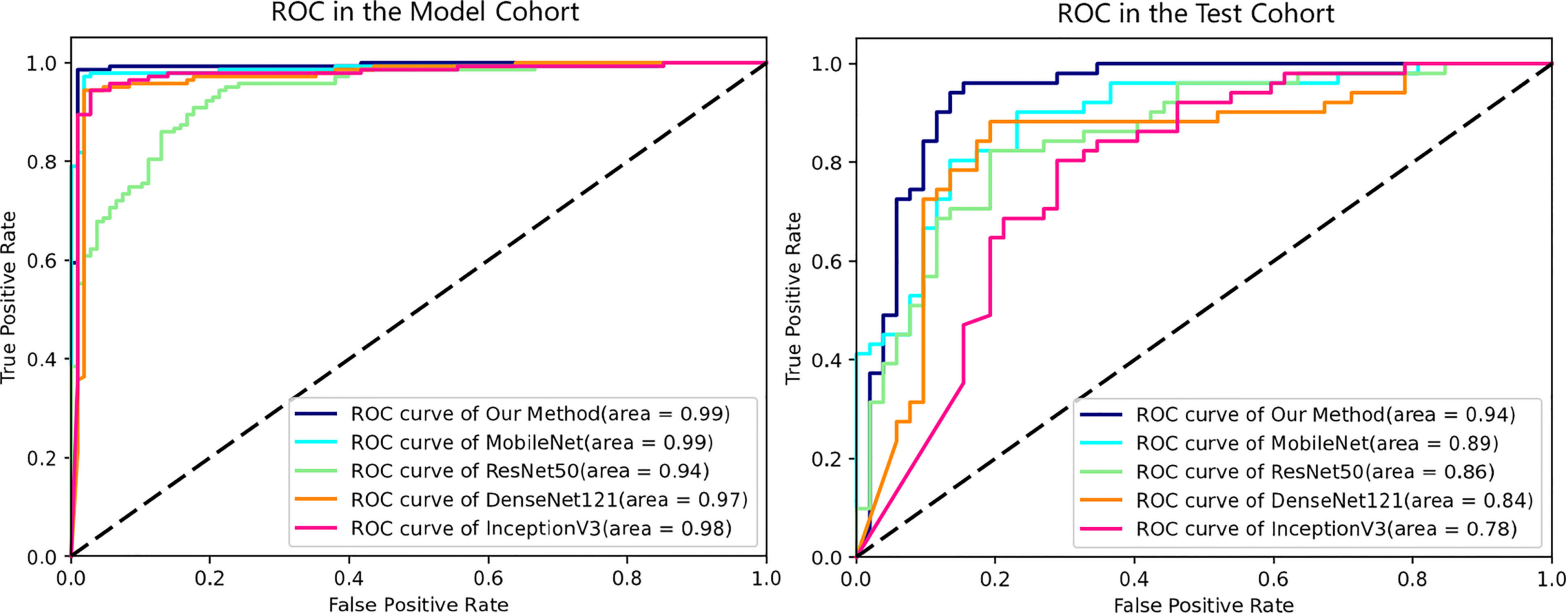
ROC curves of all deep learning models in the model and test cohorts. All methods showed excellent AUCs in model cohort, while the ROC curves in test cohort reflect the diagnosis pressure of lesions in similar clinical backgrounds on different DL methods. ROC, receiver operating characteristic; AUC, area under the ROC curve; DL, deep learning.

In the model cohort, our proposed method and the other baselines all showed great diagnostic power (AUCs of 100%, 100%, 100%, 100%, and 96.00% for our method, MobileNet, Resnet50, DenseNet121, and InceptionV3, respectively) (**Figure 5**), while in the test cohort of cases with a similar clinical background, only our proposed method had the highest diagnostic power in differentiating difficult cases (**Figure 5**). This result also reflected higher diagnostic pressure in cases with a similar clinical background. Depicted in **Table 2** are the results of the diagnostic power of all the methods in the test cohort.

**Table 2:** Diagnostic performance of all deep learning models in the test cohort.

Diagnostic index	Our method	MobileNet	Resnet50	DenseNet121	InceptionV3
AUC	93.68%	89.06%	85.67%	83.94%	78.13%
95% CI upper	98.77%	95.33%	92.93%	92.32%	87.27%
95% CI lower	88.60%	82.80%	78.41%	75.55%	68.99%
Sensitivity	96.08%	96.08%	88.24%	88.24%	92.16%
Specificity	76.92%	61.54%	59.62%	61.54%	53.85%
Accuracy	86.41%	78.64%	73.79%	74.76%	72.82%
F1-score	88.66%	81.67%	76.78%	77.59%	77.05%
PPV	80.33%	71.01%	68.18%	69.23%	66.20%
NPV	95.24%	94.12%	83.78%	84.21%	87.50%
FPR	23.08%	38.46%	40.38%	38.46%	46.15%
FNR	3.92%	3.92%	11.76%	11.76%	7.84%

AUC, area under the receiver operating characteristic curve; PPV, positive predictive value; NPV, negative predictive value; FPR, false-positive rate; FNR, false-negative rate.

### Diagnostic Robustness of Proposed Model

To avoid sample error and to evaluate the robustness of all DL methods, 5-fold cross-validation was performed among the models we used (**Table 3**). The results showed satisfactory robustness of our proposed model.

**Table 3:** Accuracy of all models in 5-fold cross-validation.

Accuracy	Xception	MobileNet	Resnet50	DenseNet121	InceptionV3
Training cohort					
Accuracy1	98.26%	94.77%	83.06%	95.89%	95.39%
Accuracy2	98.13%	94.27%	84.56%	95.39%	94.89%
Accuracy3	98.63%	94.89%	84.06%	95.89%	96.14%
Accuracy4	98.01%	94.52%	83.94%	96.26%	94.89%
Accuracy5	98.01%	95.15%	84.70%	96.14%	95.77%
Validation cohort					
Accuracy1	98.00%	94.53%	88.06%	96.02%	95.52%
Accuracy2	98.51%	96.52%	82.09%	98.01%	97.51%
Accuracy3	96.52%	94.03%	84.08%	96.02%	92.54%
Accuracy4	99.00%	95.52%	84.58%	94.53%	97.51%
Accuracy5	99.00%	93.00%	81.50%	95.00%	94.00%

Models in 5-fold cross-validation were capped at 50 epochs. Our proposed Xception method showed best robustness compared to other baselines.

## Discussion

In this study, we built a DL model fully dedicated to quickly identifying HCC from FLL in high-risk patients solely based on B-mode US images, and our study showed a promising result of AUC of 93.68%. To add more credibility, the data from 407 patients in our study were all referred to surgery pathological results.

Among the global major guidelines (3, 4, 6, 7, 22), semiannual AFP and US have been recommended for the population at risk of developing HCC. However, AFP is negative in nearly two-thirds of patients at any stage of HCC (9). A systemic review reported that the pooled sensitivities for early-stage HCC detection with US and AFP were 63% and dramatically dropped to 45% with US only (10). Among benign FLLs, FNH is the second most common benign FLL with a prevalence of 0.9%–3% in the adult population (16, 23). Unlike most hemangioma presenting classic characteristics, more than 60% of FNH cases appear hypoechoic, making it difficult to differentiate from HCC only on B-mode US in HBV-infected patients (13, 14, 16). According to the US LI-RADS, an FLL with a size over 10 mm in patients at risk for developing HCC will be categorized as US-3, in which a further examination is recommended. For those solely found FLLs without elevated AFP, advanced imaging modalities or invasive examination will be needed for further information, but they are time-consuming, have a high cost, have a risk of complications, and are limited by medical resources. Due to cost-effectivity concerns, numerous HCC risk score systems for different etiologies, antivirus status, or with/without cirrhosis have been proposed to increase the yield of HCC detection (24–28), while the screening method with US has not been changed for HCC surveillance. For patients with a higher risk of HCC, an easy and effective way to improve current US screening performance is an urgent requirement.

DL in US could provide an innovative approach to identify malignancy in clinical surveillance in a quick, non-invasive, and reliable way. With its breakthrough in recent years, AI has evolved various techniques including ML and DL. As the state-of-the-art ML approach, DL has attracted more attention in the field of medicine, as it has shown promising results using more complex algorithms to simulate the work of the human brain.

ML/DL based on US images has been reported to have good performance in roughly differentiating benignity and malignancy (19, 20) and is increasingly adopted in recent studies focusing on histological subtype differentiation (29–31).

Xi et al. trained the model based on 596 patients, which achieved an accuracy of 84% in distinguishing roughly malignant and benign hepatic tumors (20). But the composition of the FLLs was not described in their study; moreover, 331 patients among the 596 were confirmed by MRI. A generalized utilization is limited, as it was not referred to histological results.

Qin at el. developed a B-mode US-based radiomics model to determine the histological origin of liver metastasis (30). Three 2-classification models were built for distinguishing digestive tract vs. non-digestive tract tumors, breast cancer vs. non-breast cancer, and lung cancer vs. other malignancies. Similarly, Peng et al. built two models to distinguish subtypes of primary hepatocellular carcinoma, which are HCC-vs.-non-HCC model and ICC-vs.-combined-HCC-ICC (29). However, given the nature of the two-classifier of conventional ML method, differentiating subtypes among 3 types of FLLs will be complicated (32). The aforementioned studies used the conventional ML method by building a series of models to repeat the comparison procedure in order to determine subtypes of FLLs. In the testing cohort, both studies showed moderate AUC for each model (0.728–0.775), considering that as each diagnosis process goes through two to three tandem models, the accuracy for differential diagnosis of subtypes might not be ideal. A multicenter study used over 150,000 images focused on the differentiation of FLL subtypes, including cyst, hemangioma, HCC, and liver metastasis (33). With help of a huge sample of FLLs, the model based on the DL method in their study is able to do multi-grouping tasks, and diagnosis performance for every subtype was achievable. The overall accuracy of 89.1% for four discrimination was achieved (33).

In differentiating between HCC and FNH, Nie et al. enrolled 156 cases (101 HCC vs. 55 FNH cases) to establish a radiomics model based on CT images, achieving an AUC of 0.917 to distinguish HCC from FNH (34). In their study, they used traditional radiomics methods, which require hand-operated feature extraction from input images, while DL method applied in our study learns these features automatically and directly from inputs. What is more, we collected cases in similar clinical backgrounds (all with past HBV infection and no elevated AFP) as a test set to make the best of image information, achieving an AUC of 93.34% in the test set. This end-to-end workflow and higher accuracy accelerate the process in a reliable way, making it easier to integrate into the current clinical diagnosis workflow.

Recent studies by Li et al. also developed models for differentiating HCC from FNH, but the data were based on contrast-enhanced US (CEUS) (31). Considering the current high accuracy of CEUS in diagnosing HCC and FNH, space for improvement by DL is limited. While considerable improvement is made by DL solely on B-mode US images, a much more generalized utilization is also feasible because of the widespread use of conventional US.

The application of AI to image diagnosis has two main requirements—large data and the specific situation of the application. Given the rich varieties of FLLs, a reasonably applied situation of the DL method should be specialized. Our DL model managed to identify AFP-negative HCC from benign FLL in HBV-infected patients during surveillance. This specialized usage of US-based DL could be a potential additional workup in the current diagnosis and surveillance strategy of HCC screening. On the other hand, the need for large data is also met by the enormous data generated on screening of HCC in a big population with high risk.

Our ideal aim is to build a computer-aided diagnosis (CAD) tool to assist in identifying HCC on first-line US surveillance; from this point, we acknowledge the following limitations in our study. Firstly, regenerative nodule (RN) or dysplastic nodule (DN) are also common in patients under HCC surveillance; it is said that RN is detectable in 25% of cirrhotic livers (35). Therefore, it is necessary to add those FLLs that usually share similar features with HCC in US morphology and clinical background. External validation from other institutions was lacking since this study was a single-center study; to avoid bias and verify the generalization ability, a multicenter study with a larger sample and more FLL types including RN and DN is necessary.

In conclusion, this study suggests that our DL approach has great potential to assist B-mode US in identifying AFP-negative HCC from FLL found in surveillance of HBV-infected patients.

## Data Availability Statement

The datasets presented in this study can be found in online repositories. The names of the repository/repositories and accession number(s) can be found below: https://github.com/Size-Hou/Deep-Learning-for-Approaching-an-HCC-hepatic-cell-carcinoma-screening-dilemma.

## Ethics Statement

The studies involving human participants were reviewed and approved by Ethics Committee of the Zhongshan Hospital of Fudan University. The patients/participants provided their written informed consent to participate in this study.

## Author Contributions

W-BZ and S-ZH equally contributed. W-BZ and Y-LC contributed to this paper with conception and design. W-BZ and Y-LC collected the data. FM and YD performed ultrasound scans and image interpretation. S-ZH and J-GC performed image analysis and construction of the deep learning model. W-BZ drafted the manuscript. W-PW contributed to the revision and the critical idea of this paper. All authors approved the final submitted version.

## Funding

The study was supported by the National Natural Science Foundation of China (Grant no. 82071924), Natural Science Foundation Project of Shanghai (Grant no. 20ZR1452800), Clinical Research Plan of Shanghai Shengkang Hospital Development Center (Grant no. SHDC2020CR1031B), and Shanghai Municipal Key Clinical Specialty (Grant no. shslczdzk03501).

## Conflict of Interest

The authors declare that the research was conducted in the absence of any commercial or financial relationships that could be construed as a potential conflict of interest.

## Publisher’s Note

All claims expressed in this article are solely those of the authors and do not necessarily represent those of their affiliated organizations, or those of the publisher, the editors and the reviewers. Any product that may be evaluated in this article, or claim that may be made by its manufacturer, is not guaranteed or endorsed by the publisher.

## Abbreviations

HCC, hepatocellular carcinoma; AFP, α-fetoprotein; AI, artificial intelligence; ML, machine learning; DL, deep learning; CAD, computer-aided diagnosis; FLL, focal liver lesion; FNH, focal nodular hyperplasia; HBV, hepatitis B virus; AUC, area under the receiver operating characteristic curve; PPV, positive predictive value; NPV, negative predictive value; FPR, false-positive rate; FNR, false-negative rate; APASL, Asian Pacific Association for the Study of the Liver; EASL, European Association for the Study of the Liver; AASLD, American Association for the Study of Liver Diseases; JSH, Japan Society of Hepatology; US, ultrasound.
